# 3,5,6-Trimethyl­thieno[2,3-*d*]pyrimidin-4(3*H*)-one

**DOI:** 10.1107/S1600536812035416

**Published:** 2012-08-23

**Authors:** Khamroqul Khatamov, Fozil Saitqulov, Jamshid Ashurov, Khusnutdin Shakhidoyatov

**Affiliations:** a A. Navoiy Samarkand State University. University Avenue 15, Samarkand, Uzbekistan; b Institute of Bioorganic Chemistry, Academy of Sciences of Uzbekistan, H. Abdullaev Str. 83, Tashkent 100125, Uzbekistan; c S. Yunusov Institute of the Chemistry of Plant Substances, Academy of Sciences of Uzbekistan, H. Abdullaev Str. 83, Tashkent 100125, Uzbekistan

## Abstract

In the title compound, C_9_H_10_N_2_OS, the thienopyrimidine ring system is almost planar [greatest deviation from the mean plane = 0.0318 (13) Å for the S atom]. The crystal packing features C—H⋯O hydrogen bonds and π–π stacking inter­actions between inversion-related pairs of mol­ecules with a centroid–centroid distance of 3.530 (3) Å.

## Related literature

For the synthesis, properties and biological activity of pyrim­idinone derivatives, see: Litvinov (2004 ▶); Al-Taisan *et al.* (2010 ▶). For the crystal and mol­ecular structures of related compounds, see: Tashkhodzhaev *et al.* (2002 ▶).
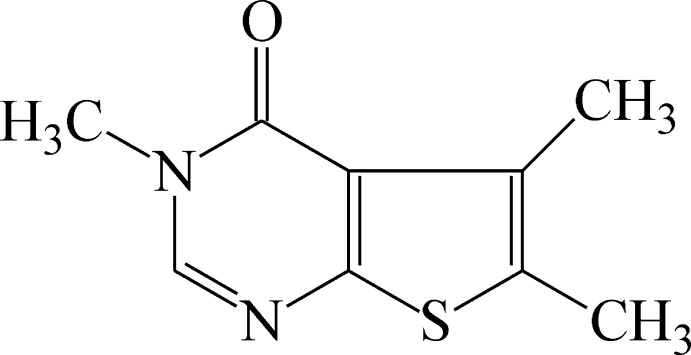


## Experimental

### 

#### Crystal data

C_9_H_10_N_2_OS
*M* *_r_* = 194.25
Monoclinic, 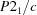

*a* = 8.027 (3) Å
*b* = 10.706 (5) Å
*c* = 10.907 (3) Å
β = 97.333 (3)°
*V* = 929.7 (6) Å^3^
*Z* = 4
Cu *K*α radiation
μ = 2.77 mm^−1^
*T* = 293 K
0.42 × 0.36 × 0.28 mm

#### Data collection

Oxford Diffraction Xcalibur Ruby diffractometer
Absorption correction: multi-scan (*CrysAlis PRO*; Oxford Diffraction, 2009 ▶) *T*_min_ = 0.324, *T*_max_ = 1.000
2983 measured reflections
1588 independent reflections
1208 reflections with *I* > 2σ(*I*)
*R*_int_ = 0.029

#### Refinement

*R*[*F* ^2^ > 2σ(*F* ^2^)] = 0.050
*wR*(*F* ^2^) = 0.151
*S* = 1.05
1588 reflections
122 parameters
H-atom parameters constrained
Δρ_max_ = 0.27 e Å^−3^
Δρ_min_ = −0.22 e Å^−3^

### 

Data collection: *CrysAlis PRO* (Oxford Diffraction, 2009 ▶); cell refinement: *CrysAlis PRO*; data reduction: *CrysAlis PRO*; program(s) used to solve structure: *SHELXS97* (Sheldrick, 2008 ▶); program(s) used to refine structure: *SHELXL97* (Sheldrick, 2008 ▶); molecular graphics: *XP* in *SHELXTL* (Sheldrick, 2008 ▶); software used to prepare material for publication: *SHELXL97*.

## Supplementary Material

Crystal structure: contains datablock(s) I, global. DOI: 10.1107/S1600536812035416/aa2064sup1.cif

Structure factors: contains datablock(s) I. DOI: 10.1107/S1600536812035416/aa2064Isup2.hkl

Supplementary material file. DOI: 10.1107/S1600536812035416/aa2064Isup3.cml

Additional supplementary materials:  crystallographic information; 3D view; checkCIF report

## Figures and Tables

**Table 1:** Hydrogen-bond geometry (Å, °)

*D*—H⋯*A*	*D*—H	H⋯*A*	*D*⋯*A*	*D*—H⋯*A*
C2—H2⋯O1^i^	0.93	2.32	3.250 (4)	173

Symmetry code: (i) 
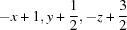
.

**Fig. 1.**
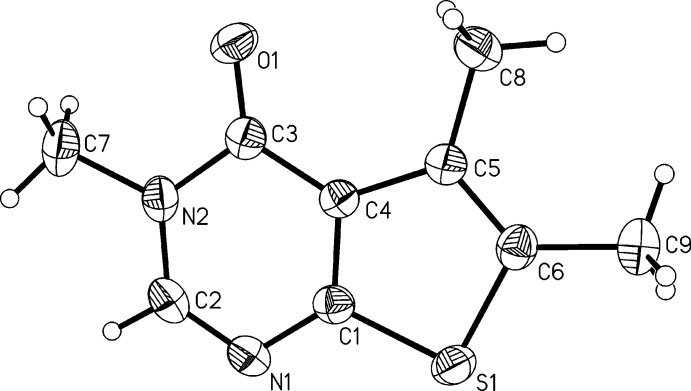
The molecular structure of the title compound, showing 30% probability displacement ellipsoids and the atom numbering scheme.

**Fig. 2.**
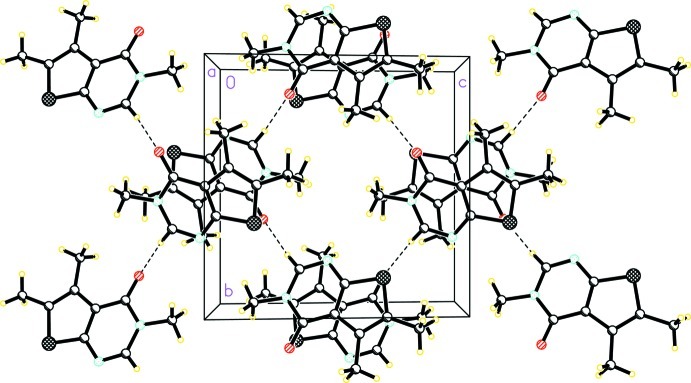
A packing diagram for title compound. C—H···O hydrogen bonds are shown as dashed lines.

## supplementary crystallographic information

### Comment

The derivatives of thienopyrimidine are of interest because of their interesting pharmacological and biological activities (Litvinov, 2004). The title compound, C_9_H_10_N_2_OS, may be used for obtaining bioactive molecules. The asymmetric unit of the title compound consists of a single molecule (Fig.1). The thienopyrimidine ring system (N1/C2/N3/C4/C5/C6/C7/C8/S1) is ideal planar with greatest deviation from mean plane 0.0318 (12) Å for the S1). The crystal packing is stabilized by intermolecular hydrogen bonds (Table 1) and π-π stacking interactions between inversion-related pair of molecules with a centroid-centroid (N1/C2/N3/C4/C5/C6/C7/C8/S1) distance of 3.530 (3) Å.

### Experimental

To a suspension of 5,6-trimethylthieno(2,3 - d)pyrimidin-4-one (181 mg, 0.1 mmol) in 50 ml e thanol sodium hydride (24 mg, 1 mmol) was added. The mixture was stirred at room temperature for 30 min. Then a solution of methyl iodide(142 mg, 1 mmol) in ethanol was added drop wise. The solution was stirred at 353–363 K for 4 h, then the solution was evaporated under reduced pressure and the residue was treated by distilled water. The precipitate was filtered and dried. Yield of 84% (174 mg). Crystals suitable for single-crystal X-ray diffraction were obtained by recrystallization from chloroform at room temperature.

### Refinement

All H atoms were placed in geometrically idealized positions (C—H 0.96 (methyl) and C—H 0.93 Å (phenyl) and treated as riding on their parent atoms, with U(H) set to 1.2 to 1.5U(C).

### Crystal data

**Table:**

C_9_H_10_N_2_OS	*F*(000) = 408
*M**_r_* = 194.25	*D*_x_ = 1.388 Mg m^−^^3^
Monoclinic, *P*2_1_/*c*	Cu *K*α radiation, λ = 1.54184 Å
Hall symbol: -P 2ybc	Cell parameters from 185 reflections
*a* = 8.027 (3) Å	θ = 4.1–43.7°
*b* = 10.706 (5) Å	µ = 2.77 mm^−^^1^
*c* = 10.907 (3) Å	*T* = 293 K
β = 97.333 (3)°	Block, colourless
*V* = 929.7 (6) Å^3^	0.42 × 0.36 × 0.28 mm
*Z* = 4	

### Data collection

**Table:**

Oxford Diffraction Xcalibur Ruby diffractometer	1588 independent reflections
Radiation source: fine-focus sealed tube	1208 reflections with *I* > 2σ(*I*)
Graphite monochromator	*R*_int_ = 0.029
Detector resolution: 10.2576 pixels mm^-1^	θ_max_ = 67.2°, θ_min_ = 5.6°
ω scans	*h* = −8→9
Absorption correction: multi-scan (*CrysAlis PRO*; Oxford Diffraction, 2009)	*k* = −12→12
*T*_min_ = 0.324, *T*_max_ = 1.000	*l* = −13→11
2983 measured reflections	

### Refinement

**Table:**

Refinement on *F*^2^	Secondary atom site location: difference Fourier map
Least-squares matrix: full	Hydrogen site location: inferred from neighbouring sites
*R*[*F*^2^ > 2σ(*F*^2^)] = 0.050	H-atom parameters constrained
*wR*(*F*^2^) = 0.151	*w* = 1/[σ^2^(*F*_o_^2^) + (0.1022*P*)^2^ + 0.0132*P*] where *P* = (*F*_o_^2^ + 2*F*_c_^2^)/3
*S* = 1.05	(Δ/σ)_max_ < 0.001
1588 reflections	Δρ_max_ = 0.27 e Å^−^^3^
122 parameters	Δρ_min_ = −0.22 e Å^−^^3^
0 restraints	Extinction correction: *SHELXL97* (Sheldrick, 2008), Fc^*^=kFc[1+0.001xFc^2^λ^3^/sin(2θ)]^-1/4^
Primary atom site location: structure-invariant direct methods	Extinction coefficient: 0.0070 (14)

### Special details

**Table:**

Geometry. All s.u.'s (except the s.u. in the dihedral angle between two l.s. planes) are estimated using the full covariance matrix. The cell s.u.'s are taken into account individually in the estimation of s.u.'s in distances, angles and torsion angles; correlations between s.u.'s in cell parameters are only used when they are defined by crystal symmetry. An approximate (isotropic) treatment of cell s.u.'s is used for estimating s.u.'s involving l.s. planes.
Refinement. Refinement of *F*^2^ against ALL reflections. The weighted *R*-factor *wR* and goodness of fit *S* are based on *F*^2^, conventional *R*-factors *R* are based on *F*, with *F* set to zero for negative *F*^2^. The threshold expression of *F*^2^ > σ(*F*^2^) is used only for calculating *R*-factors(gt) *etc*. and is not relevant to the choice of reflections for refinement. *R*-factors based on *F*^2^ are statistically about twice as large as those based on *F*, and *R*- factors based on ALL data will be even larger.

### Fractional atomic coordinates and isotropic or equivalent isotropic displacement parameters (Å^2^)

**Table:**

	*x*	*y*	*z*	*U*_iso_*/*U*_eq_	
C1	0.3074 (3)	0.1156 (2)	0.4761 (3)	0.0448 (7)	
C2	0.4524 (4)	0.1684 (3)	0.6555 (3)	0.0559 (8)	
H2	0.5198	0.2239	0.7052	0.067*	
C3	0.3198 (3)	−0.0372 (3)	0.6401 (2)	0.0467 (7)	
C4	0.2664 (3)	−0.0020 (2)	0.5153 (2)	0.0396 (6)	
C5	0.1683 (3)	−0.0728 (2)	0.4196 (2)	0.0446 (7)	
C6	0.1362 (4)	−0.0066 (3)	0.3125 (2)	0.0498 (7)	
C7	0.4735 (4)	0.0352 (4)	0.8380 (3)	0.0693 (10)	
H7A	0.3808	0.0084	0.8791	0.104*	
H7B	0.5195	0.1109	0.8754	0.104*	
H7C	0.5584	−0.0285	0.8454	0.104*	
C8	0.1125 (4)	−0.2045 (3)	0.4345 (3)	0.0615 (8)	
H8A	0.0263	−0.2062	0.4879	0.092*	
H8B	0.2062	−0.2539	0.4699	0.092*	
H8C	0.0690	−0.2379	0.3551	0.092*	
C9	0.0413 (4)	−0.0454 (4)	0.1917 (3)	0.0694 (9)	
H9A	−0.0690	−0.0091	0.1831	0.104*	
H9B	0.0319	−0.1348	0.1890	0.104*	
H9C	0.0998	−0.0173	0.1254	0.104*	
N1	0.4025 (3)	0.2028 (2)	0.5443 (2)	0.0537 (6)	
N2	0.4153 (3)	0.0576 (2)	0.7075 (2)	0.0505 (6)	
O1	0.2922 (3)	−0.13487 (19)	0.69116 (18)	0.0595 (6)	
S1	0.22443 (10)	0.14187 (7)	0.32561 (6)	0.0543 (3)	

### Atomic displacement parameters (Å^2^)

**Table:**

	*U*^11^	*U*^22^	*U*^33^	*U*^12^	*U*^13^	*U*^23^
C1	0.0458 (16)	0.0418 (14)	0.0482 (15)	0.0049 (11)	0.0116 (12)	−0.0006 (11)
C2	0.0553 (19)	0.0501 (17)	0.062 (2)	0.0007 (13)	0.0055 (14)	−0.0163 (14)
C3	0.0443 (16)	0.0495 (16)	0.0468 (15)	0.0075 (12)	0.0074 (12)	−0.0022 (12)
C4	0.0368 (14)	0.0398 (14)	0.0430 (14)	0.0064 (10)	0.0081 (11)	0.0000 (10)
C5	0.0427 (16)	0.0448 (15)	0.0470 (15)	0.0027 (11)	0.0083 (12)	−0.0010 (11)
C6	0.0476 (17)	0.0553 (17)	0.0458 (15)	0.0063 (13)	0.0036 (12)	−0.0013 (12)
C7	0.073 (2)	0.088 (3)	0.0436 (17)	0.0157 (18)	−0.0037 (15)	−0.0115 (15)
C8	0.067 (2)	0.0505 (17)	0.067 (2)	−0.0105 (14)	0.0080 (16)	−0.0035 (14)
C9	0.072 (2)	0.083 (2)	0.0512 (18)	−0.0023 (18)	−0.0023 (15)	−0.0075 (16)
N1	0.0568 (15)	0.0428 (13)	0.0613 (16)	0.0001 (11)	0.0071 (12)	−0.0060 (11)
N2	0.0519 (14)	0.0564 (15)	0.0419 (13)	0.0091 (11)	0.0006 (10)	−0.0089 (10)
O1	0.0688 (15)	0.0553 (13)	0.0531 (12)	0.0013 (10)	0.0025 (10)	0.0164 (9)
S1	0.0631 (6)	0.0502 (5)	0.0493 (5)	0.0040 (3)	0.0068 (3)	0.0099 (3)

### Geometric parameters (Å, º)

**Table:**

C1—N1	1.365 (4)	C6—C9	1.494 (4)
C1—C4	1.382 (4)	C6—S1	1.739 (3)
C1—S1	1.714 (3)	C7—N2	1.461 (4)
C2—N1	1.282 (4)	C7—H7A	0.9600
C2—N2	1.364 (4)	C7—H7B	0.9600
C2—H2	0.9300	C7—H7C	0.9600
C3—O1	1.218 (3)	C8—H8A	0.9600
C3—N2	1.419 (4)	C8—H8B	0.9600
C3—C4	1.424 (4)	C8—H8C	0.9600
C4—C5	1.441 (3)	C9—H9A	0.9600
C5—C6	1.362 (4)	C9—H9B	0.9600
C5—C8	1.495 (4)	C9—H9C	0.9600
			
N1—C1—C4	126.3 (3)	N2—C7—H7C	109.5
N1—C1—S1	122.1 (2)	H7A—C7—H7C	109.5
C4—C1—S1	111.6 (2)	H7B—C7—H7C	109.5
N1—C2—N2	125.8 (3)	C5—C8—H8A	109.5
N1—C2—H2	117.1	C5—C8—H8B	109.5
N2—C2—H2	117.1	H8A—C8—H8B	109.5
O1—C3—N2	119.6 (3)	C5—C8—H8C	109.5
O1—C3—C4	127.9 (3)	H8A—C8—H8C	109.5
N2—C3—C4	112.4 (2)	H8B—C8—H8C	109.5
C1—C4—C3	118.9 (2)	C6—C9—H9A	109.5
C1—C4—C5	112.6 (2)	C6—C9—H9B	109.5
C3—C4—C5	128.5 (2)	H9A—C9—H9B	109.5
C6—C5—C4	112.0 (2)	C6—C9—H9C	109.5
C6—C5—C8	123.8 (3)	H9A—C9—H9C	109.5
C4—C5—C8	124.2 (2)	H9B—C9—H9C	109.5
C5—C6—C9	129.1 (3)	C2—N1—C1	113.8 (3)
C5—C6—S1	112.0 (2)	C2—N2—C3	122.6 (2)
C9—C6—S1	118.9 (2)	C2—N2—C7	119.2 (3)
N2—C7—H7A	109.5	C3—N2—C7	118.2 (3)
N2—C7—H7B	109.5	C1—S1—C6	91.90 (13)
H7A—C7—H7B	109.5		

### Hydrogen-bond geometry (Å, º)

**Table:**

*D*—H···*A*	*D*—H	H···*A*	*D*···*A*	*D*—H···*A*
C2—H2···O1^i^	0.93	2.32	3.250 (4)	173

Symmetry code: (i) −*x*+1, *y*+1/2, −*z*+3/2.
